# The Effect of Nutritional Mobile Apps on Populations With Cancer: Systematic Review

**DOI:** 10.2196/50662

**Published:** 2025-02-05

**Authors:** Krystal Lu Shin Ng, Murallitharan Munisamy, Joanne Bee Yin Lim, Mustafa Alshagga

**Affiliations:** 1 Division of Biomedical Sciences Faculty of Science and Engineering University of Nottingham Malaysia Campus Semenyih Malaysia; 2 National Cancer Society Malaysia Kuala Lumpur Malaysia; 3 School of Humanities Faculty of Art and Social Sciences University of Nottingham Malaysia Campus Semenyih Malaysia

**Keywords:** cancer, mobile app, nutrition, body composition, quality of life, mobile health, mHealth, diet, intervention, mobile phone, PRISMA

## Abstract

**Background:**

Limited access to nutrition support among populations with cancer is a major barrier to sustainable and quality cancer care. Increasing use of mobile health in health care has raised concerns about its validity and health impacts.

**Objective:**

This systematic review aimed to determine the effectiveness of commercial or cancer-specific nutritional mobile apps among people living with cancer.

**Methods:**

A systematic search of the CENTRAL, Embase, PubMed (MEDLINE), and Scopus databases was carried out in May 2024. All types of intervention studies were included, except observational studies, gray literature, and reference lists of key systematic reviews. Studies were eligible for inclusion if they involved (1) patients with or survivors of cancer and (2) nutrition-related mobile apps. Studies were excluded if the nutrition intervention was not delivered via mobile app or the app intervention was accompanied by dietary counseling. The review process was conducted based on the PRISMA (Preferred Reporting Items for Systematic Reviews and Meta-Analyses) guidelines. The Risk of Bias 2 and Risk of Bias in Nonrandomized Studies tools were used to assess the study quality. The Cochrane Review Manager (version 5.4) software was used to synthesize the results of the bias assessment.

**Results:**

A total of 13 interventions were included, comprising 783 adults or teenagers with cancer. Most studies focused on breast cancer (6/13, 46%), overweight (6/13, 46%), and survivors (9/13, 69%). Data on anthropometry and body composition (7/13, 54%; 387 participants), nutritional status (3/13, 23%; 249 participants), dietary intake (7/13, 54%; 352 participants), and quality of life (6/13, 46%; 384 participants) were gathered. Experimental groups were more likely to report significant improvements in body weight or composition, dietary compliance, nutritional status, and quality of life than control groups.

**Conclusions:**

Although mobile app platforms are used to deliver nutrition interventions, the evidence for long-term efficacy, particularly in populations with cancer, remains elusive. More robust randomized controlled trials with larger sample sizes, as well as more homogeneous population characteristics and outcome measures, are warranted.

**Trial Registration:**

PROSPERO CRD42023330575; https://tinyurl.com/55v56yaj

## Introduction

### Background

More than 50% of patients with cancer are likely to develop undernutrition upon diagnosis [1]. At least 5% of patients with cancer who are malnourished report drastic weight loss [2]. In total, 3 out of 5 patients report a significant weight reduction ranging from 1 to 10 kg 6 months after a cancer diagnosis [3]. Surprisingly, approximately 20% of patients with cancer die of undernutrition and its complications [4]. It is necessary to implement an early screening and detection of undernutrition based on the parameters of dietary intake, biochemical indexes, and body weight and composition. The overall nutritional status can be evaluated using cancer-specific assessment tools such as the Subjective Global Assessment, Patient-Generated Subjective Global Assessment (PG-SGA), and Mini Nutritional Assessment [5].

European Society of Parenteral and Enteral Nutrition guidelines have highlighted the importance of a multidisciplinary approach in managing undernutrition among patients with cancer [4]. However, this nutritional issue is not considered as equally important as the cancer disease itself [6,7]. If undernutrition is left untreated, this can result in poor immune response, increased treatment toxicities, impaired quality of life (QoL), increased risk of infection, increased admission rates and hospital stays, and increases in health care costs [4,7,8].

Overnutrition or excessive body fatness is another nutritional disorder that should be gaining greater attention in survivorship care [9,10]. Approximately 1 in 3 survivors of cancer report having obesity and not meeting the American Cancer Society’s BMI guidelines of <30 kg/m^2^ [10]. It is highly recommended that those living with or free of cancer eat a balanced diet to reduce the risk of recurrence and promote healthy survivorship [11].

To sustain a normal body weight, patients with cancer are advised to consume enough food to meet their daily requirement of energy and protein. In view of the differences in energy expenditure, the European Society of Parenteral and Enteral Nutrition recommends that the total energy requirement of patients with cancer be similar to that of survivors or healthy populations [4]. This elucidates that focusing on the basic principle of a balanced diet could be a nutrition guideline for patients with cancer, particularly those who are undernourished.

Studies have shown that approximately 90% of patients with cancer perceive nutrition support as an essential component in oncology care. However, less than half of patients with cancer are seen by dietitians [12]. According to the PG-SGA score, in a study by Pinho et al [1], 45% of patients with cancer required dietary intervention. In spite of that, dietetic support is not readily accessible to patients throughout their cancer journey. The high prevalence of undernutrition is commonly observed in people with upper digestive cancer, head and neck cancer, and lung cancer [1,2]. Still, in a study by Deftereos et al [7], approximately 40% of patients with upper digestive cancer did not receive any dietetic intervention before surgery.

Poor access to dietary services can be attributable to several factors, including lack of qualified dietitian staffing, lack of integration of nutrition services, lack of medical reimbursement for nutrition services, lack of awareness of cancer-related malnutrition, and inconsistent practice of nutritional risk screening in oncology [8]. Without a professional evidence-based dietary intervention, patients are likely to obtain information from the media or their peers. Conflicting information about nutrition makes them confused about what they should eat to optimize their well-being after a cancer diagnosis. Due to fear of cancer recurring, survivors can be desperate to modify their dietary habits [13,14]. This results in the adoption of unproven dietary strategies, including fad diets, juicing, and herbs and supplements, as well as avoiding certain food groups that are essential to their health [12].

The World Health Organization has called for a global initiative to leverage the use of digital health in areas of clinical medicine and public health [15]. During the COVID-19 pandemic, the application of digital technology targeting from planning and tracking, medical supplying, and screening for infection to clinical management was successful [16]. The pandemic has brought about an accelerated growth of digital health use to deliver continuous health care services while reducing virus transmission. For instance, telemedicine allowed for appointment scheduling and enhanced feasible health care delivery during the pandemic [17]. In addition, the use of digital health encourages engagement between practitioners and patients, as well as ensuring a sustainable health care system [18,19].

### Objectives

To date, the implications of mobile app use in cancer screening, prevention, and management have been greatly highlighted [20,21]. However, there is a lack of empirical evidence that focuses on populations with cancer [22-24] and mobile app platforms [22], particularly for healthy eating and nutritional management. This systematic review aimed to determine the effectiveness of commercial or cancer-specific nutritional apps in improving nutrition-related health outcomes for people receiving treatment for or living with cancer.

## Methods

### Study Protocol and Guidance

The protocol for this review was registered with PROSPERO (registration number: CRD42023330575) [25]. This review was reported based on the PRISMA (Preferred Reporting Items for Systematic Reviews and Meta-Analyses) 2020 guidelines (Multimedia Appendix 1) [26].

### Databases and Search Strategy

Systematic searches were conducted across 4 databases—CENTRAL, Embase, PubMed**,** and Scopus—in May 2024. The search strategy incorporated Medical Subject Headings (MeSH), keywords, and free-text searches that related to the 3 main concepts: mobile apps, cancer, and nutrition. The search string used in the literature search was as follows: “Mobile Applications”[Mesh] OR “mobile application*”[tw] OR “mobile apps”[tw] OR “mobile app”[tw] OR “mobile technolog*”[tw] OR “mobile health”[tw] OR mHealth[tw] OR smartphone[tw] OR “smart phone”[tw] OR telemedicine[tw] AND “Neoplasms”[Mesh] OR cancer*[tw], neoplasm*[tw] OR oncology[tw] OR tumour*[tw] OR tumor*[tw] OR malignant[tw] OR malignanc*[tw] AND “Diet, Food, and Nutrition”[Mesh] OR nutrition[tw] OR diet[tw] OR eat[tw] OR food[tw] (Multimedia Appendix 2). It included original articles published between January 2013 and December 2023 and in the English language. This is a change from the registered protocol [25].

### Study Selection

First, EndNote (version 20.3; Clarivate Analytics) was used to identify and remove duplicates from the list. The titles and abstracts of articles were screened independently by 2 reviewers (KLSN and MA) to identify potential eligible studies. The references retrieved from the search were categorized as excluded or included based on the population, intervention, comparator, outcome, and study design criteria [27]:

- Population—this included individuals who had a cancer diagnosis or a history of cancer.
- Intervention—the studies included commercial or cancer-specific mobile apps and nutrition-related key functions, including recording or monitoring food intake and providing feedback, recommendations, or coaching. Due to limited studies that included stand-alone use of mobile apps, studies on multicomponent interventions, such as targeting sleep, physical activity, or psychosocial care, were included.
- Outcome—the measures included changes in nutritional-related health outcomes. Due to a lack of feasibility studies, data on the evaluation of the quality of the mobile apps were not included. This is a change from the registered protocol [25].
- Study design—all types of intervention studies were considered, such as pretest-posttest studies, pilot studies, quasi-experimental studies, and randomized controlled trials (RCTs). Observational studies, gray literature, expert recommendations, or references in articles were not included.

A full-text screening was carried out by reviewing in detail the studies that were not excluded at the first screening based on the inclusion criteria. Each full text was retrieved and assessed independently by the same authors before inclusion in the review. Non–English-language articles were excluded. Any disagreements during the selection process were resolved through consensus.

### Data Extraction and Synthesis

The data were extracted systematically from each article by KLSN and then checked by MA. The data included were authors, publication date (year), country, study design, sample size, participant characteristics, and details on the mobile app intervention. Next, data were extracted based on the type of population (survivors or patients receiving treatment), components of the app (eg, diet alone or diet plus physical activity), duration of the intervention and follow-up, and outcome measures (body weight, body composition, QoL, and dietary factors). A comparison of the descriptive findings was made across the studies. The outcome data between groups and before and after the intervention within groups were compared using mean differences and significance values (*P* value). The heterogeneity of the interventions and measures precluded a statistical combination of the quantitative findings; therefore, a meta-analysis was not conducted.

### Risk-of-Bias Assessment

Analysis of the risk of bias was conducted using the Review Manager (version 5.4; The Cochrane Collaboration) software. The Risk of Bias 2 (RoB 2) and Risk of Bias in Nonrandomized Studies (ROBINS) tools were used for RCTs and non-RCTs, respectively. The risk of bias assessment was carried out by 2 reviewers independently (KLSN and MA). All discrepancies were resolved through consensus.

The seven areas included in the RoB 2 tool were (1) random sequence generation, (2) allocation concealment, (3) blinding of participants and personnel, (4) blinding of outcome assessment, (5) incomplete outcome data, (6) selective reporting, and (7) other bias. For the ROBINS tool, the seven areas included were (1) bias due to confounding, (2) bias in selection of participants for the study, (3) bias in classification of interventions, (4) bias due to deviations from the intended intervention, (5) bias due to missing data, (6) bias in measurement of outcomes, and (7) bias in selection of the reported results. According to the Cochrane Handbook for Systematic Reviews of Interventions, each area was assigned a classification of low, unclear, or high risk of bias [28].

## Results

### Overview

**Figure 1:**
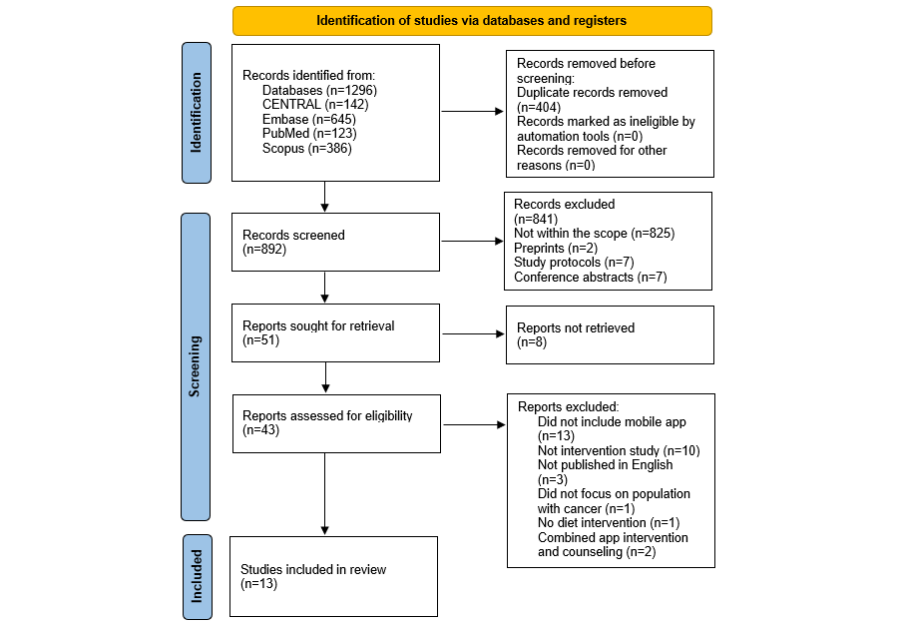
Study flowchart adapted from the PRISMA (Preferred Reporting Items for Systematic Reviews and Meta-Analyses) statement.

A total of 1296 articles were identified from all database searches. After 31.17% (404/1296) of duplicates were removed, the abstracts and titles of 68.83% (892/1296) of relevant articles were screened. The full texts of 5.7% (51/892) of these studies were retrieved and assessed for eligibility based on the population, intervention, comparator, outcome, and study design criteria. Finally, 13 articles were eligible to be included in this review. The procedure for article selection is shown in Figure 1 [26].

### Study Characteristics

#### Participants

Of the 13 included studies, there were 7 (54%) that were RCTs [29-35]; 4 (31%) that were single-arm, pretest-posttest studies [36-39]; and 2 (15%) that were quasi-experimental studies [40,41]. In total, 62% (8/13) of the included studies were conducted in the United States [29-31,35-38,40]; 15% (2/13) were conducted in South Korea [33,39]; and the remaining 23% (3/13) were conducted in Germany [41], Turkey [32], and Australia [34]. Most studies (9/13, 69%) were published within the past 5 years [29,30,32-35,38-40].

Among the 13 studies, a total of 783 participants with cancer aged 12 to 75 years was included. The sample sizes ranged from 22 to 127. In total, 15% (2/13) of the studies had no comparison groups [36,37]. A total of 8% (1/13) of the studies were conducted on teenagers [38], whereas the remaining 92% (12/13) of the studies were conducted on adults aged between 18 and 75 years. The most prevalent condition targeted in the studies was breast cancer (6/13, 46%) [29-32,37,40], followed by gastrointestinal cancer (2/13, 15%) [33,34], hematologic cancer (2/13, 15%) [35,38], mixed cancer (2/13, 15%) [36,41], and esophageal cancer (1/13, 8%) [39]. A total of 46% (6/13) of the studies were conducted among participants with overweight or obesity [29,35-38,40]. In total, 15% (2/13) of the studies were conducted among participants with body weight within the normal range [33,39], whereas 38% (5/13) of the studies did not state the weight status of the population [30-32,34,41]. Of the 13 studies, 9 (69%) recruited survivors [29-32,35-38,40], and the remaining 4 (31%) recruited patients with newly diagnosed cancer or receiving treatment [33,34,39,41]. Table 1 shows the summary of the study details, participant characteristics, and intervention types.

**Table 1:** Study characteristics.

Study and country	Study design	Sample size, N	Population characteristics	Cancer type	Intervention	Control	App features
Chow et al [35], 2020, United States	Pilot RCT^a^	Experimental group: 24; control group: 17	Adults diagnosed for ≥5 years; mean age 44 (range 20.9-54.0) years in the experimental group and 46.0 (range 20.2-54.8) years in the control group; mean BMI 28.6 (SD 6.5) kg/m^2^ in the experimental group and 29.6 (SD 6.3) kg/m^2^ in the control group	Hematologic	Had access to HealthWatch 360 (GB HealthWatch) and Fitbit Flex wristband (Google) with goal setting and peer support	Had access to Fitbit Flex wristband and HealthWatch 360 without goal setting and peer support	Commercial app; tracking of food intake
McCarroll et al [36], 2015, United States	Pretest-posttest study	50	Women with OW^b^ or obesity diagnosed over the previous 3 years; mean age 58.4 (SD 10.3) years	Endometrium or breast cancer	Had access to LoseIt!	—^c^	Commercial app; logging of food, exercise, and BW^d^ and provision of personalized feedback
Orlemann et al [41], 2018, Germany	Pilot; QED^e^	Experimental group: 12; control group: 12	Adults receiving treatment; mean age 58.4 (range 27-90) years	Mixed (GI^f^ tumor; n=16)	Had access to OncoFood (Huawei Technologies Co Ltd)	Received nutrition counseling and therapy without app	Custom-developed app; recording of food intake and monitoring of nutritional goals and BW
Stubbins et al [37], 2018, United States	Prospective, single arm, and open label	33	Survivors with OW; mean age 57 (SD 9) years; mean BMI 32.7 (SD 5.7) kg/m^2^	Breast cancer	Used MOCHA^g^ for ≥5 days	—	Custom-developed app; access to sleep and mood data, provision of a list of cardiovascular and strength activities with amount of calories burned, logging of food, and monitoring of progress
Baik et al [30], 2020, United States	Pilot RCT	Experimental group: 39; control group: 39	Latina survivors; mean age 52.54 (SD 11.36) years	Breast cancer	Access to My Guide	Access to My Health	Custom-developed app; My Guide: focus on ways to cope with side effects of treatment, stress management, social support, and breast cancer–related knowledge; My Health: provides recommendations regarding nutrition, exercise, and prevention of chronic illnesses
Buscemi et al [31], 2019, United States	Pilot RCT	Experimental group: 40; control group: 40	Latina survivors; mean age 52.54 (SD 11.36) years	Breast cancer	Access to My Guide	Access to My Health	Custom-developed app; My Guide: focus on ways to cope with side effects of treatment, stress management, social support, and breast cancer–related knowledge; My Health: provides recommendations regarding nutrition, exercise, and prevention of chronic illnesses
Cairo et al [40], 2020, United States	Non-RCT	Experimental group: 66; control group: 61	Female survivors; mean age 51.4 (SD 8.1) years in the experimental group and 56.7 (SD 9.8) years in the control group; mean BMI 29.4 (SD 6.0) kg/m^2^ in the experimental group and 30.2 (SD 7.3) kg/m^2^ in the control group	Breast cancer	Access to Vida	Received self-guided nutrition “toolkit,” exercise stretch band, pedometer, and self-guided walking DVD	Commercial app; tracking of medication, diet, exercise, sleep, and BW and pairing with a certified coach
Fuemmeler et al [38], 2020, United States	Single-group pretest-posttest design	15	Teenage survivors; mean age 14.8 (SD 1.97) years; mean BMI 22.6 (SD 4.1) kg/m^2^ in the experimental group and 22.7 (SD 2.7) kg/m^2^ in the control group (data from post hoc analysis)	Acute lymphoblastic leukemia or lymphoma	Used Mila Blooms for ≥4 weeks	Used Mila Blooms for <4 weeks	Custom-developed app; monitors progress, allows for autonomic feedback, and uses game mechanics to promote healthy eating and PA^h^
Allicock et al [29], 2021, United States	Pilot RCT	Experimental group: 13; control group: 9	African American women after treatment (except Herceptin therapy and endocrine pills); mean age 52.8 (SD 9.57) years in the experimental group and 51.44 (SD 9.18) years in the control group; mean BMI 33.26 (SD 5.42) kg/m^2^ in the experimental group and 38.35 (SD 7.08) kg/m^2^ in the control group	Breast cancer	Access to CHAT^i^ and ActiGraph wGT3X-BT accelerometer plus tailored health messages	Access to CHAT and ActiGraph wGT3X-BT accelerometer	Custom-developed app; provision of suggestions about PA and healthy diet
Çınar et al [32], 2021, Turkey	Single-blinded, single-center, randomized pretest-posttest design	Experimental group: 31; control group: 33	Women receiving hormonal therapy; mean age 45.7 (SD 9.0) years	Breast cancer	Received routine care plus mobile app–based training	Received routine care	The nature of the app was not mentioned; provision of information about breast cancer, symptom diary, balanced diet, regular PA, and stress management
Keum et al [33], 2021, South Korea	Prospective, single-center, nonblinded RCT	Experimental group: 20; control group: 20	Patients scheduled for chemotherapy; median age 62 (range 45-70) years in the experimental group and 61 (range 34-78) years in the control group; mean BMI 21.91 (SD 1.57) kg/m^2^ in the experimental group and 23.5 (SD 2.72) kg/m^2^ in the control group	Pancreatic cancer	Access to Noom mobile app (Noom Inc)	Did not have access to the Noom app and received none of the nutrition intervention	Commercial app; logging of food, step count, and BW; provided coaching and allowed for messaging for tracking caloric intake and muscle gain
Yang et al [39], 2021, South Korea	Prospective, single-arm pilot study	Experimental group: 38; control group: 60	Men scheduled for neoadjuvant chemoradiotherapy; median age 59.2 (SD 6.5) years in the experimental group and 58.5 (SD 7.8) years in the control group; mean BMI 21.8 (SD 2.6) kg/m^2^ in the experimental group and 22 (SD 6) kg/m^2^ in the control group	Esophageal cancer	Access to Noom mobile app	Previous cohort: received usual care	Commercial app; recording, monitoring, and provision of recommendations about diet, exercise, and BW changes
Huggins et al [34], 2022, Australia	3-arm RCT	Mobile app group: 36; telephone group: 38; control group: 37	Adults newly diagnosed with cancer; mean age 63.2 (SD 9.9) years in the control group, 67.5 (SD 10.3) years in the telephone group, and 66.6 (SD 9.7) years in the mobile app group; mean BW 75.0 (SD 20.0) kg in the control group, 71.9 (SD 12.7) kg in the telephone group, and 76.4 (SD 14.7) kg in the mobile app group	Upper GI cancer	Mobile app group: received symptom-directed nutrition intervention via the internet-enabled mobile app “myPace”; telephone group: received symptom-directed nutrition intervention via telephone	Received usual care	Commercial app; self-monitoring of goal attainment and BW

^a^RCT: randomized controlled trial.

^b^OW: overweight.

^c^Not applicable.

^d^BW: body weight.

^e^QED: quasi-experimental design.

^f^GI: gastrointestinal.

^g^MOCHA: Methodist Hospital Cancer Health Application.

^h^PA: physical activity.

^i^CHAT: Creating Healthy Actions Through Technology.

#### Mobile Apps

##### Types

Most studies (10/13, 77%) included a multicomponent intervention that combined diet with physical activity, psychosocial support, sleep, or behavior modification. Specifically, 50% (5/10) of these studies involved a combination of diet and physical activity [29,35,36,38,39], with additional components in the other 50% (5/10) of the studies [30-32,37,40]. The remaining 23% (3/13) of the studies included a dietary intervention as a single component [33,34,41].

##### Duration

The duration of the interventions ranged from 4 weeks to 6 months. A total of 62% (8/13) of the studies lasted up to 8 weeks [29-31,36-39,41], with 75% (6/8) of these studies including anthropometry or body composition as outcome measures. A total of 23% (3/13) of the studies lasted between 12 and 16 weeks [32,33,35], with one of the studies mainly evaluating QoL. The remaining 15% (2/13) of the studies lasted up to 6 months [34,40] and included both anthropometry and QoL measures. A total of 38% (5/13) of the studies continued to evaluate the participants’ progress after the intervention by investigating changes in QoL or dietary intake [29-31,34,35].

##### Features

A total of 46% (6/13) of the studies included the common features of logging, tracking, or monitoring in the mobile apps [33-35,37,38,41]. In total, 31% (4/13) of the studies focused on the provision of dietary information [29-32], whereas the remaining 23% (3/13) of the studies allowed for logging and provision of guidance or coaching [36,39,40]. Table 1 provides a more detailed description.

### Retention Rate

Of the 13 studies, 9 (69%) reported the percentage of participants who remained in the study over the intervention or follow-up periods. A total of 44% (4/9) of these studies reported a retention rate of >90% [29,31,35,40], 44% (4/9) reported retention rates of 70% to 90% [33,36,37,39], and 11% (1/9) reported a retention rate of <70% [34]. Table 2 provides a more detailed description.

**Table 2:** Key findings of the included studies.

Study			App intervention duration		Follow-up		Retention rate (%)		Outcome measures		Main findings
**Diagnosis**											
	Huggins et al [34], 2022	18 weeks		30 weeks		49.5		QALYs^a^ (EQ-5D-5L tool) QoL^b^ (EORTC QLQ-C30^c^ scale) Nutritional status (PG-SGA^d^–Short Form) Self-reported BW^e^		Mean weight 75.6 (SD 20.3) kg at 3 months, 75.6 (SD 17.5) kg at 6 months, and 73.2 (SD 18.4) kg at 12 months in the control group; 71.7 (SD 11.8) kg at 3 months, 70.2 (SD 11.7) kg at 6 months, and 68.6 (SD 13.3) kg at 12 months in the telephone group; and 71.7 (SD 15.6) kg at 3 months, 68.7 (SD 14.1) kg at 6 months, and 68.5 (SD 14.1) kg at 12 months in the mobile app group; *P*=.08 for control group vs telephone group; *P*=.03 for mobile app group vs telephone group; *P*=.48 for mobile app group vs control group Mean QoL score 54.3 (SD 25.1) at 3 months, 69.8 (SD 12.2) at 6 months, and 72.2 (SD 15.9) at 12 months in the control group; 66.4 (SD 19.7) at 3 months, 68.0 (SD 28.13) at 6 months, and 74.8 (SD 23.8) at 12 months in the telephone group; and 62.3 (SD 24.5) at 3 months, 59.25 (SD 21.1) at 6 months, and 73.5 (SD 20.5) at 12 months in the mobile app group; *P*=.22 for control group vs telephone group; *P*=.08 for mobile app group vs telephone group; *P*=.85 for mobile app group vs control group Mean QALY score 0.55 (SD 0.28) at 12 months in the control group, 0.57 (SD 0.28) at 12 months in the telephone group, and 0.59 (SD 0.23) at 12 months in the mobile app group; *P*>.99 for control group vs telephone group; *P*=.71 for mobile app group vs telephone group; *P*=.14 for mobile app group vs control group Mean PG-SGA score 7.5 (SD 5.0) at 3 months, 4.6 (SD 3.6) at 6 months, and 4.1 (SD 4.1) at 12 months in the control group; 7.8 (SD 5.7) at 3 months, 6.2 (SD 5.1) at 6 months, and 4.3 (SD 4.7) at 12 months in the telephone group; and 8.4 (SD 6.1) at 3 months, 7.2 (SD 4.0) at 6 months, and 4.9 (SD 3.6) at 12 months in the mobile app group; *P*=.35 for control group vs telephone group; *P*=.58 for mobile app group vs telephone group; *P*=.19 for mobile app group vs control group	
**Treatment**											
	Orlemann et al [41], 2018	4 weeks		—^f^		NR^g^		BW, BMI, SMM^h^, and FFM^i^ (BIA^j^) Nutritional goals (intake of protein, fibers, energy, carbohydrates, and fats)		Mean change in BW 1.03 kg in the experimental group and –1.46 kg in the control group (*P*=.045) Mean change in SMM 0.58 kg in the experimental group and –0.61 kg in the control group (*P*=.009); mean change in FFM after the intervention (*P*=.03) *P*=.91 for difference in mean changes in the intake of protein and fats, *P*=.34 for difference in mean changes in the intake of fiber, *P*=.27 for difference in mean changes in the intake of carbohydrates, and *P*=.42 for difference in mean changes in the intake of energy in the control group after the intervention; mean values NR	
	Çınar et al [32], 2021	12 weeks		—		NR		QoL (FACT-ES^k^) Symptom distress (NCCN^l^ Distress Thermometer)		QoL: t_30_=–5.13 and *P*<.001 in the experimental group and t_32_=3.25 and *P*=.003 in the control group; physical well-being: t_30_=–4.60 and *P*<.001 in the experimental group and t_32_=1.13 and *P*=.27 in the control group; emotional well-being: t_30_=–2.58 and *P*=.02 in the experimental group and t_32_=2.88 and *P*=.007 in the control group; functional well-being: t_30_=–1.01 and *P*=.32 in the experimental group and t_32_=2.67 and *P*=.01 in the control group; endocrine symptoms: t_30_=–6.49 and *P*<.001 in the experimental group and t_32_=3.08 and *P*=.004 in the control group; pretest distress score: 1003 (*P*=.32); posttest distress score: –2265 (*P*=.03)	
	Keum et al [33], 2021	12 weeks		—		82.5		QoL (EORTC QLQ-C30) Nutritional status (PG-SGA) SMI^m^ (CT^n^) Total protein and energy intake		Reduced SMI: –3.27 in the experimental group and –13.96 in the control group (*P*=.11) Improved GHS^o^ and QoL in experimental group compared to control group (*P*=.004) Mean protein intake after the intervention: 1.3 g per kg per day in the experimental group and 1 g per kg per day in the control group (*P*=.02); mean energy intake after the intervention: 25.2 kcal per kg per day in the experimental group and 17.7 kcal per kg per day in the control group (*P*=.04) Improved PG-SGA score in both groups (*P*<.001)	
	Yang et al [39], 2021	8 weeks		—		78.9		SMI (CT) NLR^p^, PLR^q^, and PNI^r^		Mean change in SMI after the intervention –7.4% (SD 6.5%) in the experimental group and –8.1% (SD 5.3%) in the control group (*P*=.57) PNI: mean change –9.8 (SD 6) in the experimental group and –6.7 (SD 7.5) in the control group (*P*=.04); NLR: mean change 0.4 (SD 3.9) in the experimental group and 0.6 (SD 5.1) in the control group (*P*=.82); PLR: mean change 84.1 (SD 157.6) in the experimental group and 62.4 (SD 173.4) in the control group (*P*=.55)	
**Survivorship**											
	Chow et al [35], 2020	16 weeks		8 weeks		90.2		PA^s^ Daily percentage of added sugar, saturated fat, and sodium (HEI^t^-2015) Physical health and mental health (PROMIS^u^ Global Health–10) Health-related self-efficacy score		Physical health: mean 2.7 (95% CI 0.7-4.6) in the experimental group and 1.8 (95% CI –0.3 to 3.8) in the control group (between-group *P*=.52); mental health: mean 4.2 (95% CI 1.5-6.9) in the experimental group and 1.8 (95% CI –1.1 to 4.8) in the control group (between-group *P*=.24) HEI-2015 score: mean 1.6 (95% CI –1.5 to 4.6) in the experimental group and 0.6 (95% CI –2.8 to 4.0) in the control group (between-group *P*=.67); daily percentage of added sugar: mean –0.8 (95% CI –2.2 to 0.5) in the experimental group and 0.1 (95% CI –1.5 to 1.6) in the control group (between-group *P*=.39); daily percentage of saturated fat: mean –0.3 (95% CI –1.5 to 0.9) in the experimental group and –0.8 (95% CI –2.2 to 0.6) in the control group (between-group *P*=.60); sodium intake: mean –832 (95% CI –1421 to –243) mg per day in the experimental group and –279 (95% CI –937 to 379) mg per day in the control group (between-group *P*=.22)	
	McCarroll et al [36], 2015	4 weeks		—		70		BW, BMI, and WC^v^ QoL and self-efficacy (FACT-G^w^ and Weight Efficacy Lifestyle Questionnaire) Minutes spent in PA Weekly intake of carbohydrates, fats, protein, fiber, and calories		Mean pretest BW 97.3 (SD 22.5) kg and mean posttest BW 95.0 (SD 22.1) kg (*P*<.001); mean pretest BMI 36.4 (SD 8.1) kg/m^2^ and mean posttest BMI 35.6 (SD 8.0) kg/m^2^ (*P*<.001); mean pretest WC 106.6 (SD 16.8) cm and mean posttest WC 103.4 (SD 17.4; *P*<.001) cm Mean pretest FACT-G score 50.47 (SD 13.3) and mean posttest FACT-G score 44.35 (SD 19.9; *P*=.15) Carbohydrates: mean pretest intake 120.6 (SD 69.3) g and mean posttest intake 124.0 (SD 120.3) g (*P*=.73); fat: mean pretest intake 44.1 (SD 23.4) g and mean posttest intake 58.2 (SD 60.0) g (*P*=.18); protein: mean pretest intake 55.2 (SD 26.6) g and mean posttest intake 65.4 (SD 62.3) g (*P*=.23); fiber: mean pretest intake 11.0 (SD 6.3) g and mean posttest intake 13.3 (SD 13.6) g (*P*=.28); calories: mean pretest intake 1022.6 (SD 494.4) kcal and mean posttest intake 1281.1 (SD 1130.6) kcal (*P*=.26)	
	Stubbins et al [37], 2018	4 weeks		—		75.8		Adherence to the MOCHA^x^ app System Usability Scale score Weight loss Dietitian-participant interaction		Mean reduced BW 2 (range +4 to –10.6) lbs after the intervention; *P* value NR	
	Baik et al [30], 2020	6 weeks		2 weeks		NR		QoL (FACT-B^y^) Symptom burden (25-item Breast Cancer Prevention Trial questionnaire) Cancer-specific distress (15-item Impact of Event Scale) Cancer-relevant self-efficacy (CASE-Cancer^z^) Breast cancer knowledge (16-item Knowledge About Breast Cancer questionnaire)		Experimental group—breast cancer well-being score for low app users: mean pretest score 23.47 (range 12-36) and mean posttest score 26.13 (range 14-35); control group—social well-being, score: mean pretest score 20.74 (range 5-28) and mean posttest score 22.52 (range 11-28); *P* value NR	
	Buscemi et al [31], 2019	6 weeks		2 weeks		>90		Daily intake of fat and FV^aa^ PA level		Fat sources: EMM^ab^ 2.38 (SE 0.21) in the experimental group and 2.86 (SE 0.21) in the control group at baseline, 2.42 (SE 0.22) in the experimental group and 2.38 (SE 0.21) in the control group at 6 weeks, and 2.36 (SE 0.22) in the experimental group and 2.20 (SE 0.22) in the control group at 8 weeks (*P*=.03)	
	Cairo et al [40], 2020	6 months		—		100		BW and BMI PA level Adherence to a healthy diet (27-item “Rate Your Plate” survey) Presence and severity of fatigue (VAS^ac^-Fatigue) Depression and anhedonia (PHQ^ad^ tool)		Mean reduced BW 1.8 (SD 4.9) kg in the experimental group (*P*<.01) and –0.2 (SD 3.7) kg in the control group (*P*=.70); mean reduced BMI 0.7 (SD 1.8) kg/m^2^ in the experimental group (*P*<.01) and –0.7 (SD 1.4) kg/m^2^ in the control group (*P*=.68) Mean reduced fatigue score 1.2 (SD 2.4) in the experimental group (*P*<.001) and 0.65 (SD 2.3) in the control group (*P*=.03); *P*=.36 for depression between experimental and control groups Improved adherence to a plant-based diet: mean change in score –6.2 (SD 5.8) in the experimental group (*P*<.001) and –2.0 (SD 6.5) in the control group (*P*=.02)	
	Fuemmeler et al [38], 2020	8 weeks		—		NR		Height, BW, BMI, z score, and percentile Intake of calories and nutrients PA level Diet and PA self-efficacy (PACE^ae^) User satisfaction and narrative engagement		Mean pretest BMI 22.6 (SD 4.1) kg/m^2^ and mean posttest BMI 22.8 (SD 4.1) kg/m^2^ in the experimental group (*P*=.41); mean pretest BMI 22.7 (SD 2.7) kg/m^2^ and mean posttest BMI 23.1 (SD 2.6) kg/m^2^ in the control group (*P*=.24) Mean pretest sweet food intake 8.4% (SD 3.6%) of kcal and mean posttest sweet food intake 13.5% (SD 9%) of kcal in the experimental group (*P*=.12) and mean pretest sweet food intake 8.8% (SD 6.3%) of kcal and mean posttest sweet food intake 7.5% (SD 4.8%) of kcal in the control group (*P*=.35; between-group *P*=.049); mean pretest sugary beverage intake 206.5 (SD 202.1) g and mean posttest sugary beverage intake 156.6 (SD 145.0) g in the experimental group (*P*=.08) and mean pretest sugary beverage intake 336.8 (SD 367.7) g and mean posttest sugary beverage intake 370.4 (SD 410.9) g in the control group (*P*=.04; between-group *P*=.04); mean pretest FV self-efficacy score 4.2 (SD 0.8) and mean posttest FV self-efficacy score 4.3 (SD 0.6) in the experimental group (*P*=.35) and mean pretest FV self-efficacy score 4.0 (SD 0.8) and mean posttest FV self-efficacy score 4.0 (SD 0.6) in the control group (*P*=.24; between-group *P*=.80)	
	Allicock et al [29], 2021	4 weeks		4 weeks		100		BMI and WC FV intake and percentage of energy from fat and fiber PA level		Mean change in BMI –0.19 (SD 0.35) kg/m^2^ in the experimental group (*P*=.10) and –0.24 (SD 0.76) kg/m^2^ in the control group (*P*=.76); mean WC change –1.04 (SD 0.95) cm in the experimental group (*P*=.003) and –0.47 (SD 1.57) cm in the control group (*P*=.39) Mean FV change 0.67 (SD 2.35) servings in the experimental group (*P*=.34) and 0.78 (SD 2.48) servings in the control group (*P*=.38); mean fast food intake change –1.5 (SD 1.98) servings in the experimental group (*P*=.008) and –1.11 (SD 1.45) servings in the control group (*P*=.09)	

^a^QALY: quality-adjusted life year.

^b^QoL: quality of life.

^c^EORTC QLQ-C30: European Organisation for Research and Treatment of Cancer Quality of Life Questionnaire.

^d^PG-SGA: Patient-Generated Subjective Global Assessment.

^e^BW: body weight.

^f^Not applicable.

^g^NR: not reported.

^h^SMM: skeletal muscle mass.

^i^FFM: fat-free mass.

^j^BIA: bioimpedance analysis.

^k^FACT-ES: Functional Assessment of Cancer Therapy–Endocrine Symptoms.

^l^NCCN: National Comprehensive Cancer Network.

^m^SMI: skeletal muscle index.

^n^CT: computed tomography.

^o^GHS: global health status.

^p^NLR: neutrophil-to-lymphocyte ratio.

^q^PLR: platelet-to-lymphocyte ratio.

^r^PNI: prognostic nutritional index.

^s^PA: physical activity.

^t^HEI: Healthy Eating Index.

^u^PROMIS: Patient-Reported Outcomes Measurement Information System.

^v^WC: waist circumference.

^w^FACT-G: Functional Assessment of Cancer Therapy–General.

^x^MOCHA: Methodist Hospital Cancer Health Application.

^y^FACT-B: Functional Assessment of Cancer Therapy–Breast.

^z^CASE-Cancer: Communication and Attitudinal Self-Efficacy Scale for Cancer.

^aa^FV: fruits and vegetables.

^ab^EMM: estimated marginal mean.

^ac^VAS: Visual Analog Scale.

^ad^PHQ: Patient Health Questionnaire.

^ae^PACE: Patient-Centered Assessment and Counseling for Exercise.

### Risk-of-Bias Assessment

**Figure 2:**
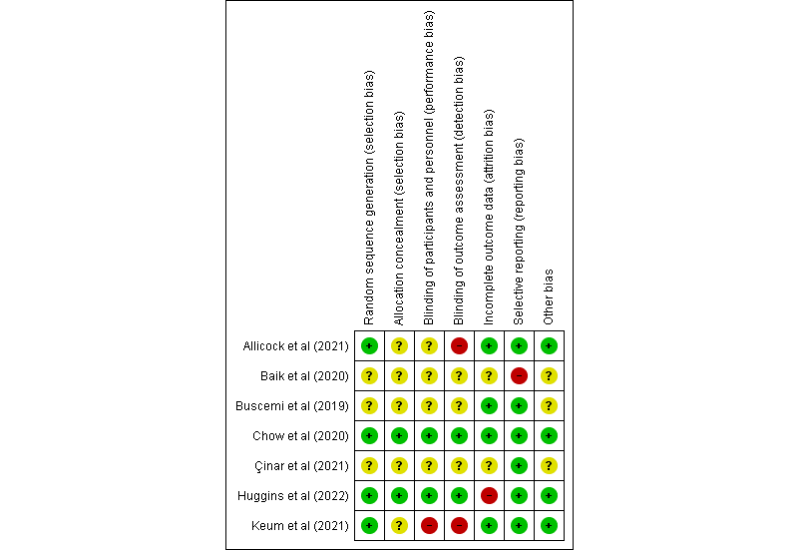
Risk-of-bias assessment of randomized controlled trials (n=7) using the Risk of Bias 2 tool, with a quality rating of low risk (–), high risk (+), or unclear risk (?).

The assessment of risk of bias was conducted for each study. The RoB 2 assessment is shown in Figure 2 [29-35]. In total, 8% (1/13) of the studies had a low risk of bias in all aspects [35]. A total of 15% (2/13) of the studies were reported as double blind [34,35]. Due to uncertainty or unblinded treatment allocation, the quality of 38% (5/13) of the trials was considered low with regard to performance and detection bias [29-33]. There was an unclear risk of selection bias in these 5 trials due to limited information about allocation concealment [29-33] and generation of a randomized sequence [30-32]. Huggins et al [34] reported a low retention rate (<50%), with the use of the multiple imputation approach for handling missing data. The suboutcomes resulting from a web-based intervention were not reported in a breast cancer study investigating the effect of mobile app–based training on QoL [32]. One study did not report the *P* value for the difference in breast cancer well-being after the intervention [30].

**Figure 3:**
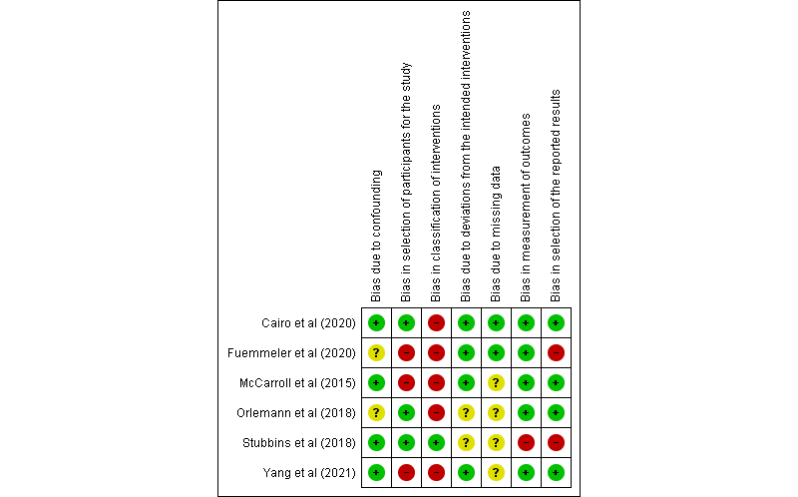
Risk-of-bias assessment using the Risk of Bias in Nonrandomized Studies tool in 6 studies, with a quality rating of low risk (–), high risk (+), or unclear risk (?).

The ROBINS assessment is shown in Figure 3 [36-41]. A total of 17% (1/6) of the studies mentioned the frequency of mobile app use (at least 5 days) during the intervention [37], whereas the remaining 83% (5/6) of the studies did not report the intervention status. In total, 67% (4/6) of the studies reported a low retention rate or uncertainty about missing data management [36,37,39,41]. A total of 50% (3/6) of the studies had a high risk of bias in the selection of study participants, which could affect the quality of the intervention and outcomes [36,38,39]. In total, 33% (2/6) of the studies did not provide information on whether there was a deviation from the intended intervention [37,41]. Fuemmeler et al [38] failed to show the changes in weight and height measurements after the intervention. A total of 17% (1/6) of the studies did not provide information about *P* values of weight loss data [37]. In total, 33% (2/6) of the studies reported no information on whether any confounding factors were present [38,41]. A total of 17% (1/6) of the studies had a high risk of bias in outcome measurements that resulted from inappropriate methods of delivering the intervention [37] and measuring outcomes [42].

### Outcome Measures

A summary of outcome measures and study findings can be found in Table 2.

#### Anthropometry and Body Composition

Of the 13 studies, 7 (54%) analyzed anthropometry measures, including body weight, BMI [29,34,36-38,40,41], and waist circumference [29,36]. Of these 7 studies, 5 (71%) intended to support weight reduction [29,36-38,40], and 2 (29%) supported weight retention [34,41]. Of the 5 studies supporting weight reduction, 2 (40%) reported significant improvement in weight after the intervention [36,40]. On the other hand, only 50% (1/2) of the studies that supported weight retention reported significant weight gain in patients with cancer who were at risk of malnutrition [41]. Huggins et al [34] reported attenuation of weight loss in patients with upper gastrointestinal cancer who received a symptom-directed nutrition intervention via telephone compared to a mobile app. A total of 29% (2/7) of the studies did not find significant changes in BMI between groups [29,38]. A study showed a decrease in weight among survivors of breast cancer with overweight; however, neither the *P* value nor the significance of the change was stated [37]. A total of 29% (2/7) of the studies reported a significant reduction in waist circumference after the intervention [29,36].

In total, 23% (3/13) of studies aiming to combat cancer-induced malnutrition assessed body composition, namely, skeletal muscle mass, fat-free mass, fat mass, and bone mineral density [33,39,41]. Significant increases in skeletal muscle mass and fat-free mass were reported in app users based on the results of bioimpedance analysis [41]. However, the studies by Keum et al [33] and Yang et al [39] did not show significant results of the skeletal muscle index using computed tomography.

#### Nutritional Status or Index

Nutritional status was evaluated in 23% (3/13) of studies aiming at weight gain [33,34,39]. According to the Scored PG-SGA, Keum et al [33] reported significant improvements in nutritional status in both the experimental and control groups but with no statistically significant difference between groups. Similarly, a nonsignificant difference in PG-SGA scores in the intervention groups (delivered via telephone or mobile app) compared with the control group was reported by Huggins et al [34]. The PG-SGA score is derived from 7 domains, namely, weight, food intake, nutrition impact symptoms, functional capabilities, presence of catabolic condition, metabolic demand, and physical examination. The scores range from 0 to 53, with higher scores indicating poorer nutritional status [43].

Another study measured the prognostic nutritional index (PNI), neutrophil-to-lymphocyte ratio, and platelet-to-lymphocyte ratio for nutritional status assessment. Only the PNI showed a significant reduction in the experimental group compared to the control group [39]. These 3 indexes were derived from laboratory parameters (PNI: 10 × albumin + 0.005 × absolute lymphocyte count; neutrophil-to-lymphocyte ratio: absolute neutrophil count/absolute lymphocyte count; platelet-to-lymphocyte ratio: platelet/absolute lymphocyte count). Higher readings indicate higher level of inflammation or severity of malnutrition.

#### Dietary Factors

A total of 62% (8/13) of the studies examined the effect of nutritional mobile apps on dietary outcomes in cancer [29,31,33,35,36,38,40,41]. The common outcome measures were daily nutrient intakes [29,31,33,36,38,41] and level of adherence to dietary recommendations [35,40]. App users reported reduced consumption of high-fat food, including fast food, after the intervention [29,31]. A higher consumption of sugary beverages was observed in non–app users compared to app users, but no significant results were reported for the intake of fruits and vegetables [38]. Keum et al [33] reported higher intake of protein and energy in app users, whereas 33% (2/6) of the studies that measured daily nutrient intake did not report any significant findings [36,41].

The level of adherence to a healthy diet was analyzed in 25% (2/8) of these studies. On the basis of a Rate Your Plate survey, app users reported a significantly improved adherence to a plant-based diet [40]. However, no significant results were reported using the Healthy Eating Index score [35].

#### QoL and Symptom Burden

The impact of nutritional mobile apps on QoL was evaluated in 46% (6/13) of the studies. In total, 33% (2/6) of these studies measured QoL using the European Organisation for Research and Treatment of Cancer Quality of Life Questionnaire (EORTC QLQ-C30) [33,34]. A total of 50% (3/6) of the studies used the Functional Assessment of Cancer Therapy–General (FACT-G) [36], the Functional Assessment of Cancer Therapy–Breast (FACT-B) [30], and Functional Assessment of Cancer Therapy–Endocrine Symptoms (FACT-ES) [32]. In total, 17% (1/6) of the studies used the Patient-Reported Outcomes Measurement Information System Global Health–10 to assess QoL [35]. In total, 33% (2/6) of the studies reported significant improvements in QoL based on the EORTC QLQ-C30 [33] and FACT-ES [32] tools. Higher scores were reported for overall perception of QoL and physical, emotional, and functional well-being, whereas lower scores were reported for endocrine symptoms and psychosocial distress. The remaining studies did not report any significant QoL results [34-36,40].

The EORTC QLQ-C30 covers 5 functional domains (physical, emotional, social, role, and cognitive), 9 symptoms (fatigue, nausea and vomiting, pain, dyspnea, insomnia, appetite loss, constipation, diarrhea, and financial difficulties), and a general health perception. The total score ranges from 0 to 100, with higher scores indicating greater symptoms or better functional status [44]. The FACT-G comprises 27 items and 5 Likert rating scales (0-4), similar to the FACT-B and FACT-ES. The FACT-G measures the domains of physical, social, emotional, and functional well-being, whereas the FACT-B and FACT-ES have 11 additional breast cancer–related items and 9 additional endocrine-related items, respectively. The total score of these QoL tools can be >100, with higher scores indicating greater symptoms or better functional status [45].

The Visual Analog Scale–Fatigue and 2-item Patient Health Questionnaire were used in the study by Cairo et al [40]. Although the experimental group reported improved levels of fatigue and depression after the intervention, these changes were not statistically significant. The Visual Analog Scale–Fatigue comprises 18 items answered on a Likert scale from 0 to 10, with higher scores indicating higher levels of pain or fatigue. On the other hand, the 2-item Patient Health Questionnaire comprises 2 items pertaining to anhedonia and depression ranging from 0 to 6, with higher scores indicating more depressive symptoms [46].

No significant results were found using quality-adjusted life years (QALYs) in the study by Huggins et al [34].

## Discussion

### Principal Findings

Nutritional mobile apps for populations with cancer have the potential to improve body weight or composition, nutritional status, dietary adherence, and QoL across the continuum of cancer care. The apps offered the basic functions of recording and tracking users’ food intake and weight in general. It was unclear whether custom-developed mobile apps were efficacious for nutrition-related health outcomes and QoL in cancer care. However, incorporating commercial mobile apps seemed to be beneficial for improving nutritional care in populations with cancer. This could be due to the implementation of self-monitoring of their progress, a necessary step in delivering quality nutrition care [5]. This review observed that the beneficial effect of stand-alone interventions was comparable with that of multicomponent interventions. However, the results may not be able to discern the magnitude of the difference due to limited data. Overall, the studies included in this review were of low to moderate quality. For RCTs, lack of blinding and biased treatment allocation were the major concerns. Failure to define the intervention status in terms of types, frequency, and timing reported by non-RCTs made the evaluation of nutritional mobile apps challenging.

### Comparison to Prior Work

Among the interventions that aimed to support weight loss, almost half (2/5, 40%) reported successful weight control among patients with cancer and overweight. In public health research, the common measures are BMI, waist circumference, waist-to-hip ratio, and body fat percentage [47]. Waist-to-height ratio has also been known to be a good surrogate in predicting the risk of noncommunicable diseases [48,49]. Implementing effective dietary strategies for successful weight loss is highly recommended to reduce the risk of cancer recurrence in long-term survivorship [4]. A review underpinned the beneficial effects of eHealth interventions on weight management in survivors of cancer, with a greater impact if combined with dietary counseling [50].

Our review found reduced intake of fast food [29] and sugary food [38] in app users. When aiming at weight loss, adhering to healthy eating guidelines should be the goal to sustain good health and well-being. A bariatric study highlighted the need to change eating behaviors for sustainable weight management [51]. Self-monitoring weight changes and dietary behavior is a common feature in app-based weight loss programs. The use of mobile app interventions for improved eating behavior and diet quality seems to be promising [52]. There are multiple factors influencing eating habits among school-aged children, particularly role modeling and parenting styles [53]. In addition to app gamification, creating a conducive learning environment in schools and at home could be a way to promote healthy eating habits among children.

The primary concern regarding undernutrition is the lack of energy that the body needs to undergo cancer treatment, which could result in treatment toxicities, longer hospital stays, or reduced QoL [4]. Among interventions that aim to support weight gain in patients with cancer who were malnourished, delivering nutrition support via mobile app platforms may help prevent drastic weight loss and improve skeletal muscle mass and overall nutritional status. However, the findings of this review do not reflect the long-term beneficial effects due to lack of data. Despite the growing development of nutrition apps, tailoring dietary interventions to individuals’ needs, nutritional status, cancer type, treatment plan, and comorbid conditions is still an unmet need [54].

Of the 6 studies that focused on QoL, only 2 (33%) showed significant changes in QoL at the treatment phase based on the EORTC QLQ-C30 and FACT-ES [32,33]. A review that focused on app-based interventions to improve nutrition or lifestyle behaviors in patients with breast cancer showed a similar finding during chemotherapy [55]. This could be due to enhanced user engagement by improving self-motivation, health information, social support, and goal setting [56]. The 2 cancer-specific tools used in our review were the EORTC QLQ-C30 [44] and FACT-G [45], which allow for a multidimensional assessments of QoL. These tools yield a comprehensive evaluation of individuals’ progress. QALYs, which account for both QoL and survival, have been increasingly used as a standard measure to evaluate disease burden at the population or regional level [57,58]. However, Huggins et al [34] reported no significant results for QALYs in groups that received the intervention via mobile app or telephone compared to controls. Failure to obtain significant results could be due to less participants who continued to use the mobile app after the intervention period.

### Strengths and Limitations

This is the first review that has evaluated the impact of app-based dietary interventions in cancer care. The review was based on a systematic search strategy that focused on nutrition interventions delivered via mobile app platforms and on populations with cancer. However, this review has certain limitations. First, only English-language articles were included in the search for this review. Second, the heterogeneity of study designs, interventions, app features, and cancer types was substantial, requiring the results to be interpreted cautiously. Third, the inconsistent measurement and reporting of incomplete data made comparisons difficult across the studies. Finally, this review included pilot studies that comprised small sample sizes (11/13, 85% of the interventions enrolled <70 participants per group), resulting in limited generalizability of the study findings.

### Conclusions

Mobile app–based nutrition interventions have a favorable effect on nutritional status and QoL in patients with cancer. In addition, mobile apps that incorporate nutrition interventions could also be beneficial for survivors after cancer treatment. However, it was unclear whether custom-developed apps were efficacious for improved nutrition-related outcomes and QoL. The continuity of nutritional care in patients with cancer via mobile app platforms could help in achieving a healthy weight by improving their adherence to dietary guidelines. Although most studies yielded favorable outcomes, they were rated as being of low to moderate quality.

Future studies should emphasize randomized controlled designs, larger sample sizes, diet-only mobile apps, greater homogeneity of outcome measures and population characteristics, and high participant engagement and retention within the study.

## Appendix

Multimedia Appendix 1 PRISMA (Preferred Reporting Items for Systematic Reviews and Meta-Analyses) 2020 checklist [26].

Multimedia Appendix 2 Search strategy.

## Abbreviations

EORTC QLQ-C30: European Organisation for Research and Treatment of Cancer Quality of Life Questionnaire
FACT-B: Functional Assessment of Cancer Therapy–Breast
FACT-ES: Functional Assessment of Cancer Therapy–Endocrine Symptoms
FACT-G: Functional Assessment of Cancer Therapy–General
MeSH: Medical Subject Headings
PG-SGA: Patient-Generated Subjective Global Assessment
PNI: prognostic nutritional index
PRISMA: Preferred Reporting Items for Systematic Reviews and Meta-Analyses
QALY: quality-adjusted life year
QoL: quality of life
RCT: randomized controlled trial
RoB 2: Risk of Bias 2
ROBINS: Risk of Bias in Nonrandomized Studies
