# Supplementary figures and images for: The Effect of Nutritional Mobile Apps on Populations With Cancer: Systematic Review
Source: JMIR Cancer. 2025 Feb 5;11:e50662. doi: 10.2196/50662 (PMC11840368; doi:10.2196/50662)

Database search history

1. Pubmed


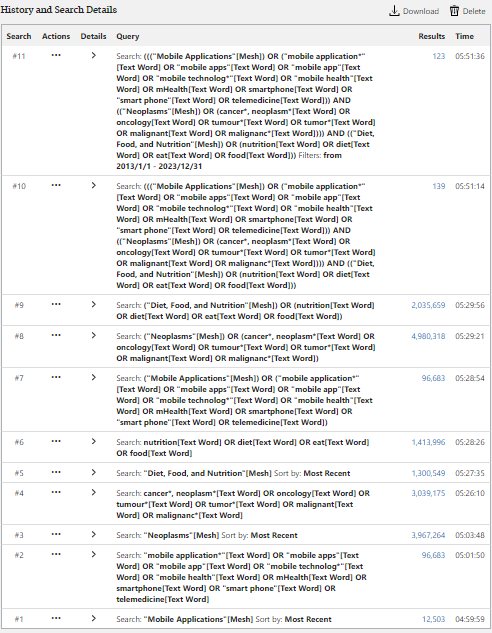


1. CENTRAL


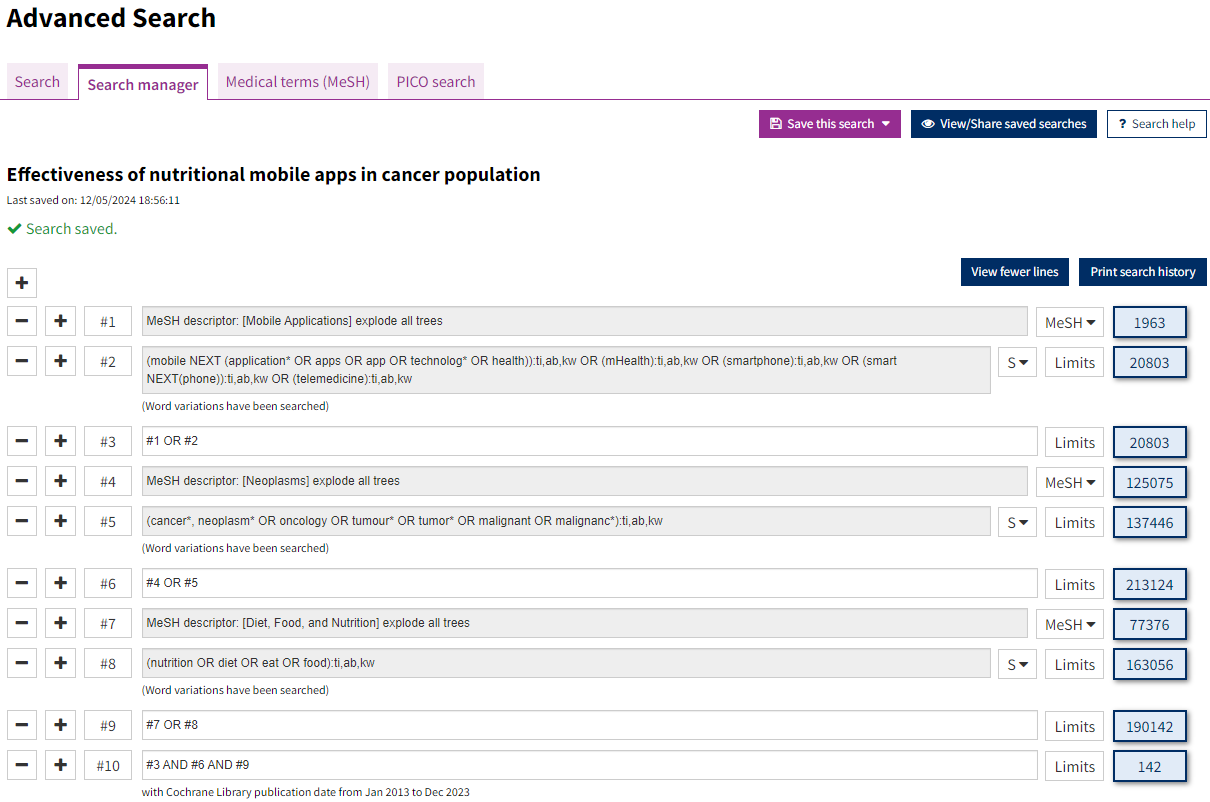


1. EMBASE


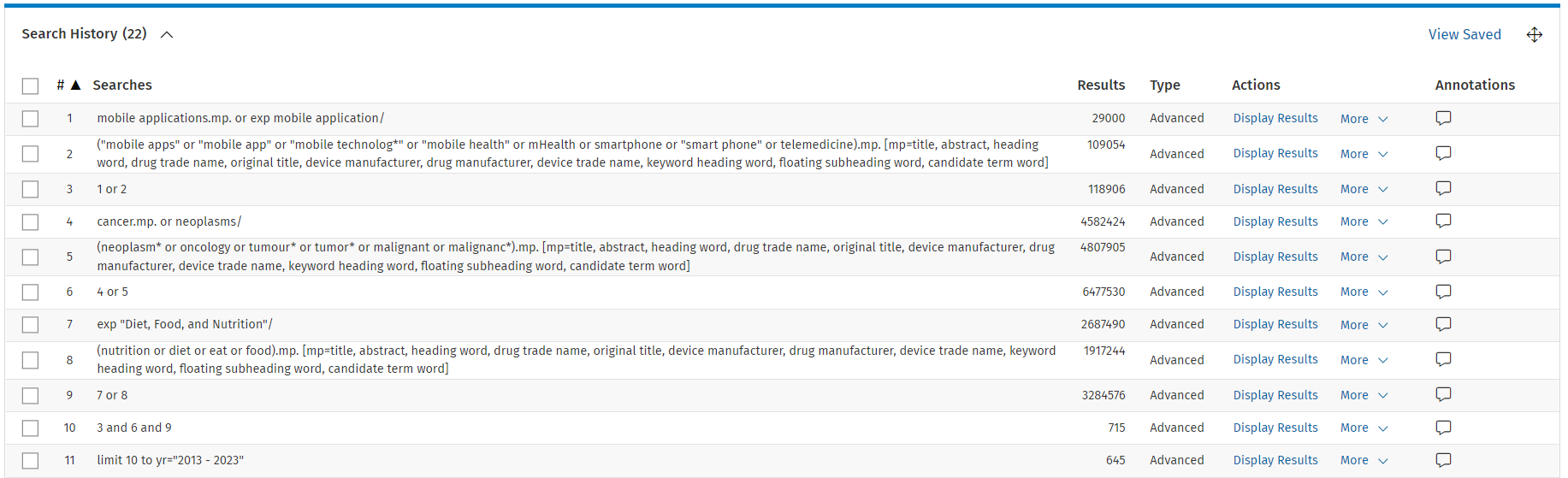


1. SCOPUS


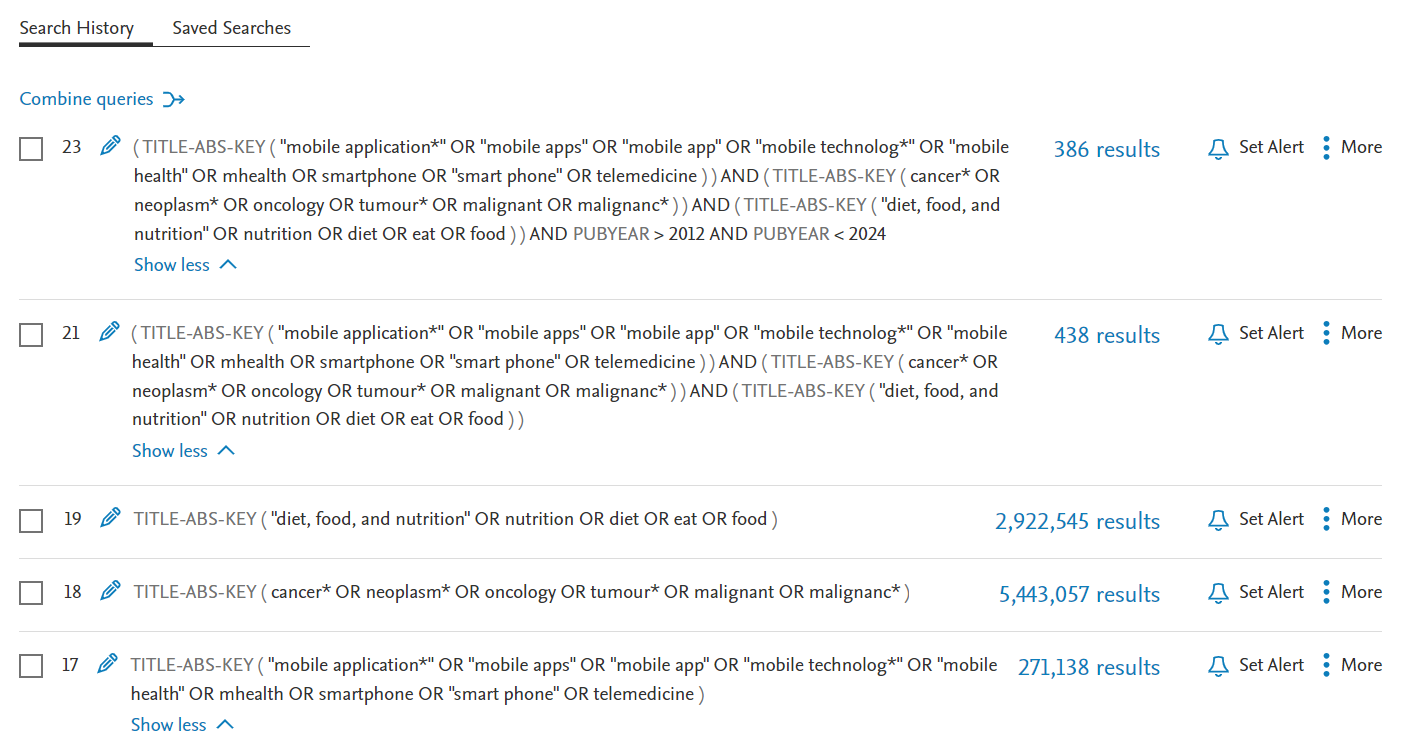

Supplement: Multimedia Appendix 2 [file cancer_v11i1e50662_app2.docx]
